# Dr. Heath’s Plan for Studying Remedies

**Published:** 1891-04

**Authors:** Alfred Heath

**Affiliations:** London, England


### DR. HEATH'S PLAN FOR STUDYING REMEDIES.

TO THE EDITORS OF THE HOMŒOPATHIC PHYSICIAN:

Allow me to call attention to an error in your notice of Dr. Wolff's "Analysis Sheets," in the February number of HOMŒOPATHIC PHYSICIAN. You say, "It is an ingenious idea and is a modification of that suggested by Dr. E. J. Lee, and of the *later device of Dr. Alfred Heath.*" I only wish to point out that mine was not the later device, but that I was the first to publish the plan which has been looked upon with great favor by large numbers of homœopaths. It will be in your memory that as long ago as the middle of 1889 I sent you a case worked on my plan, with the working, but, although you told me it was in type, it was never, for some reason, published by Dr. Lee. In January, 1890, when I saw you, it was still unpublished, and I asked you to return it to me, which you did. It was then, by the kindness of Dr. Bartlett, published in the February number of the *Hahnemannian Monthly*. In that article I mentioned that in the February number of the *Homœopathic World*, 1882, I *published* for the first time a case of mine worked on this plan; previously to that I had used the plan myself for many years, and it was the outcome of practice. I showed it before 1882 to my friend, the late Dr. David Wilson, and he expressed the highest appreciation of the plan. This year I sent you a case worked out, and at the same time I sent another case to the *Advance*. You wrote me it would take too much space, and so did not publish it, but Dr. Allen put in the plan I sent him in December's *Advance*. As I was the first to advocate and publish this plan I see no reason why Dr. Lee should have the credit of introducing it, especially as his is *really a modification of my plan*—substituting numbers for names. He has never published his in any journal. It is true that about *three or four* months ago you sent me one of his printed sheets. It was the first time I had seen it. I feel sure that the part of the notice I object to was an oversight on your part, and that you will publish this letter. I am, dear sir, yours truly,

ALFRED HEATH, M. D.

LONDON, ENGLAND, February 16th, 1891.

